# Peeling the Layers Away: The Genomic Characterization of *Bacillus pumilus* 64-1, an Isolate With Antimicrobial Activity From the Marine Sponge *Plakina cyanorosea* (Porifera, Homoscleromorpha)

**DOI:** 10.3389/fmicb.2020.592735

**Published:** 2021-01-08

**Authors:** Jéssyca Freitas-Silva, Bruno Francesco Rodrigues de Oliveira, Felipe de Mello Vigoder, Guilherme Muricy, Alan D. W. Dobson, Marinella Silva Laport

**Affiliations:** ^1^Institute of Microbiology Paulo de Góes, Federal University of Rio de Janeiro, Rio de Janeiro, Brazil; ^2^School of Microbiology, University College Cork, Cork, Ireland; ^3^Department of Genetics, Institute of Biology, Federal University of Rio de Janeiro, Rio de Janeiro, Brazil; ^4^Department of Invertebrates, National Museum, Federal University of Rio de Janeiro, Rio de Janeiro, Brazil; ^5^Environmental Research Institute, University College Cork, Cork, Ireland

**Keywords:** antimicrobials, *Bacillus pumilus*, genome mining, *Plakina cyanorosea*, sponge-associated bacteria

## Abstract

*Bacillus pumilus* 64-1, a bacterial strain isolated from the marine sponge *Plakina cyanorosea*, which exhibits antimicrobial activity against both pathogenic and drug-resistant Gram-positive and Gram-negative bacteria. This study aimed to conduct an in-depth genomic analysis of this bioactive sponge-derived strain. The nearly complete genome of strain 64-1 consists of 3.6 Mbp (41.5% GC), which includes 3,705 coding sequences (CDS). An open pangenome was observed when limiting to the type strains of the *B. pumilus* group and aquatic-derived *B. pumilus* representatives. The genome appears to encode for at least 12 potential biosynthetic gene clusters (BGCs), including both types I and III polyketide synthases (PKS), non-ribosomal peptide synthetases (NRPS), and one NRPS-T1PKS hybrid, among others. In particular, bacilysin and other bacteriocin-coding genes were found and may be associated with the detected antimicrobial activity. Strain 64-1 also appears to possess a broad repertoire of genes encoding for plant cell wall-degrading carbohydrate-active enzymes (CAZymes). A myriad of genes which may be involved in various process required by the strain in its marine habitat, such as those encoding for osmoprotectory transport systems and the biosynthesis of compatible solutes were also present. Several heavy metal tolerance genes are also present, together with various mobile elements including a region encoding for a type III-B Clustered Regularly Interspaced Short Palindromic Repeats (CRISPR) region, four prophage segments and transposase elements. This is the first report on the genomic characterization of a cultivable bacterial member of the *Plakina cyanorosea* holobiont.

## Introduction

The symbiotic relationship between marine invertebrates and microorganisms is among the oldest, most faithfully maintained, and functionally sophisticated within the realm of animal microbiomes (Petersen and Osvatic, 2018). The increased availability of -omics data continues to provide valuable information on the microbial ecology of bacterial sponge communities and their complex microbial-host interactions (Thomas et al., 2016; Moitinho-Silva et al., 2017). Nevertheless, it is also still important to isolate and genetically characterize sponge-associated bacteria to gain further insights into their role within the overall functioning of the sponge holobiont (Laport, 2018; Pita et al., 2018) as well as their potential to produce bioactive compounds with pharmaceutical applications, together with enzymes and biosurfactants with industrial utility (Santos-Gandelman et al., 2014).

From a biotechnological perspective, sponge-derived cultivable members of the phyla *Proteobacteria*, *Actinobacteria* and *Firmicutes*, such as *Pseudovibrio* (O’Halloran et al., 2011; Buijs et al., 2019), *Streptomyces* (Jackson et al., 2018; Yang et al., 2019) and *Bacillus* (Phelan et al., 2014), have been the main microbial sources targeted for the isolation and characterization of bioactive compounds. Indeed, *Bacillus* species are well-known producers of secondary metabolites, namely non-ribosomal peptides, lanthipeptides and, in particular; a wide variety of bacteriocins (Ortiz and Sansinenea, 2019). In addition, a diverse range of antibacterial and antifungal substances, together with enzymes and inhibitors, biosurfactants and compounds with bioremediation, bioleaching or cytotoxic potentials have been isolated from sponge-derived *Bacillus* (Santos-Gandelman et al., 2014; Indraningrat et al., 2016; Laport, 2018; Ortiz and Sansinenea, 2019).

Following a screening regime of bacteria isolated from the recently described marine sponge *Plakina cyanorosea* (Muricy et al., 2019), a *Bacillus pumilus* strain, identified as the number 64-1, displayed antimicrobial activity against multidrug-resistant (MDR) and medically relevant bacterial strains (Freitas-Silva et al., 2020). This motivated us to move forward with the genome sequencing of this bacterial strain to better access its potential to produce bioactive microbial metabolites. In this study, we explored the *B. pumilus* 64-1 genome isolated from *P. cyanorosea* to identify biosynthetic gene clusters (BGCs) that may be associated with its strong antimicrobial potential. Through an ecological perspective, we also explored the relevant genes potentially involved with survival in the marine environment. In addition, other biotechnological potentialities were also uncovered in this sponge-derived *Bacillus* strain.

## Materials and Methods

### Bacterial Strain

*Bacillus pumilus* 64-1 was isolated from the marine sponge *P. cyanorosea* as previously described (Freitas-Silva et al., 2020). Briefly, the sponge sample was collected manually, washed with artificial sea water [NaCl 2.34% (w/v), MgSO_4_.7H_2_O 0.49% (w/v), MgCl_2_.6H_2_O 0.4% (w/v), CaCl_2_.H_2_O 0.15% (w/v), KCl 0.075% (w/v), NaHCO_3_ 0.017% (w/v)] and immediately transferred into brain heart infusion (BHI). The sample was processed under aseptic conditions by initial maceration with glass beads. Serial dilutions (10^–3^ to 10^–5^) were prepared from sponge macerate and 100 μL aliquots of each dilution were plated in duplicate onto the BHI agar. The plates were incubated for up to seven days at 25°C and the colonies were isolated, purified, and cryopreserved at −20°C for further analysis.

### DNA Extraction

Genomic DNA was extracted from a pure culture of *B. pumilus* 64-1 following growth for 24 h on BHI medium using a modified guanidine method (Pitcher et al., 1989). These adaptations include: before the addition of lysozyme, the cells were centrifuged at 8,000 × *g* for 15 min and washed in Tris–EDTA (TE) buffer 1X (pH 8.0); thereafter, the pellet was submitted to new centrifugation at 8,000 × *g* for 5 min; and detergent polyoxyethylene lauryl ether (BRIJ) solution and chloroform-isoamyl alcohol were used instead of sarkosyl and two-propanol from the original method. The quality and purity of the DNA were checked on 0.8% (w/v) agarose gel electrophoresis, while the quantity was determined using NanoVue^TM^ Plus Spectrophotometer (GE Healthcare, United States). The extraction of plasmid DNA was performed with the Wizard^®^ Plus SV Minipreps DNA Purification System Protocol kit with lysozyme (20 mg/ml), in accordance with the manufacturer’s instructions. *Klebsiella pneumoniae* Kp13 plasmids (Ramos et al., 2014) were used as a marker of the plasmid’s molecular size. Plasmid DNA profiles were observed on 0.8% agarose gel electrophoresis with a UV transilluminator.

### Genome Sequencing and Assembly

A workflow detailing the bioinformatic tools adopted for the assembly and annotation of the *B. pumilus* 64-1 genome is presented in the Supplementary Figure 1. The fragmentation of genomic DNA and preparation of the sequencing library were achieved using a Nextera XT Sample Preparation, Nextera XT Index kits (Illumina, United States). Following purification with Agencourt AMPure XP beads (Beckman Coulter, United States) and library quantification by qPCR (KAPA-KK4824 - Illumina/Universal), whole-genome sequencing was performed on the Illumina NextSeq 500 System, with the NextSeq 500 MID Output kit (300 cycles). The quality of the generated raw paired-end reads was checked by the A5-miseq pipeline tool (Coil et al., 2015) and monitored with the FastQC program (Andrews, 2010). After trimming and the removal of adaptors/barcode sequences, *de novo* genome assembly was achieved using SPAdes 3.13.0 (Bankevich et al., 2012) and evaluated with the QUAST 5.0.2 tool (Gurevich et al., 2013). Contigs with length equal or inferior to 400 bp were removed from the scaffolds output archive. Completeness and contamination were then determined with the CheckM v1.0.18 software (Parks et al., 2015).

### Gene Prediction and Functional Annotation

The overall structural annotation was performed using the myRAST application (Overbeek et al., 2013) using reference genomes that are phylogenetically close to the genome to be annotated and the DDBJ Fast Annotation and Submission Tool (DFAST) that allows the detection of coding sequences (CDS), ribosomal (rRNA) and transporter (tRNA) RNA genes (Tanizawa et al., 2018). The translated protein sequences were submitted for functional categorization as clusters of orthologous groups (COG) within the eggNOG database (Huerta-Cepas et al., 2019). The eggNOG functional annotation was further used for validation of the explored ecological- and biotechnologically relevant genes. SignalP-4.1 (Nielsen, 2017) and TMHMM v.2.0 (Krogh et al., 2001) were chosen for the identification of signal peptides and transmembrane helices in the predicted proteins. PSORTb 3.0.2 was applied to infer their subcellular localization (Yu et al., 2010). The presence of putative plasmid sequences was carried on with PlasmidFinder 2.0 (Carattoli et al., 2014).

The identification, annotation and analysis of BGCs were performed using the antibiotics and Secondary Metabolite Analysis SHell (antiSMASH) version 5.0.0 beta (Blin et al., 2019). Moreover, the BAGEL4 webserver was used for automated identification of genes encoding for bacteriocins and (non-) bactericidal post-translationally modified peptides, with default parameters (van Heel et al., 2018).

Carbohydrate-active enzymes (CAZymes) were identified and annotated with HMMER, DIAMOND and Hotpep algorithms from the dbCAN2 metaserver (Zhang et al., 2018). HMMER webserver 2.40 based searches were performed using the phmmer parameter (Potter et al., 2018) against the MEROPS complete sequence library (Rawlings et al., 2018) to track peptidase and their inhibitors.

Groups of CDS with potential roles with survival in marine habitats, such as osmoregulatory transport systems and biosynthetic enzymes for compatible solutes (Jackson et al., 2018), were identified in the eggNOG functional annotation files. The detected transporter systems were then additionally interrogated using the Transporter Classification Database (TCDB) (Saier et al., 2016) and the Archaeal and Bacterial ABC Systems Database (ABCdb) (Fichant et al., 2006).

Acquired antimicrobial resistance genes (ARG) and/or chromosomal mutations were scanned automatically with the Resistance Gene Identifier tool (RGI 5.1.0) from the Comprehensive Antibiotic Resistance Database (CARD 3.0.7) (Alcock et al., 2020), narrowing the criteria for “perfect and strict hits only” and the sequence quality for “high quality/coverage”. Putative genes for modification or detoxification enzymes acting specifically on antimicrobials from different classes and dedicated to metal tolerance were manually identified from the eggNOG annotation results.

The identification of Clustered Regularly Interspaced Short Palindromic Repeats (CRISPRs) and *cas* genes was performed with the CRISPRCasFinder program (Couvin et al., 2018). BLASTn searches (Altschul et al., 1990) were performed with the identified direct repeats (DR) regions. The Artemis version 16.0.0 (Carver et al., 2008) was then used for the annotation and visualization of the genomic organization of the detected CRISPR regions and, finally, the RNAfold web server program was applied to reconstruct the putative secondary structures of the DR sequences (Lorenz et al., 2011). IslandViewer 4 (Bertelli et al., 2017) was employed for the integrated prediction of genomic islands, with the complete chromosomes of *B. pumilus* NCTCC 10337 (=ATCC 7061^T^) (NZ_LT906438.1) and *B. pumilus* PDSLzg-1 (NZ_CP016784.1) used as references to reorder the contigs from the *B. pumilus* 64-1 draft genome. The identification of prophage elements was performed with the PHASTER webserver (Arndt et al., 2016). Insertion sequences (ISs) were initially detected using the BLASTn searches against the ISfinder database (Siguier et al., 2006). For the additional detection of potential mobilome elements, manual curation was also performed for the localization of the keywords “phage,” “transponsase,” “integrase,” and “insertion” through the eggNOG annotation files.

### Phylogenetic Analyses

The complete 16S rRNA sequence (1,548 bp) of the *B. pumilus* 64-1 strain was recovered from its draft genome (section “Gene Prediction and Functional Annotation”). For further phylogenetic analyses, we prioritized the 16S rRNA sequences of closely related species/representatives with identity scores ≥98.7% (Rosselló-Móra and Amann, 2015) and from valid type strains from the *B. pumilus* clade: *B. pumilus* ATCC 7061^T^ (NR_043242.1), *Bacillus safensis* NBRC100820 (AB681259.1), *Bacillus zhangzhouensis* MCCC 1A05787 (JX680098.1), *Bacillus altitudinis* 41KF2b^T^ (ASJC01000029.1), *Bacillus australimaris* MCCC 1A05787 (JX680098.1), *Bacillus xiamenensis* MCCC 1A00008 (JX680066.1). The 16S rRNA gene sequences from aquatic-derived *B. pumilus* strains with deposited genome sequences (Table 1) were also retrieved from the National Center for Biotechnology Information (NCBI) GenBank database and incorporated for this analysis: *B. pumilus* 145 (CP027116.1), *B. pumilus* 150a (CP027034.1), *B. pumilus* PDSLzg-1 (CP016784.1), *B. pumilus* PE09-72 (NZ MIJA01000046.1), *B. pumilus* RI06-95 (NZ_LFGZ01000009.1), *B. pumilus* Ha06YP001 (NZ PTXV01000013.1) and *B. pumilus* SF214 (FJ977607.1).

**TABLE 1:** List of *Bacillus* genomes selected for phylogenetic analyses and the overall genome-related index (OGRIs) calculation.

Strain	Origin	Genome level^1^	Number of contigs	Genome size (Mbp)	G + C%	Accession number	References
*B. pumilus* ATCC 7061^T^	First described type strain	Scaffolds	16	3.83	41.70	ABRX00000000.1	Dodson et al. (2014)
*B. safensis* FO-36b^T^	Spacecraft and assembly-facility surfaces	Chromossome	1	3.76	41.70	ASJD00000000.1	Lai et al. (2014b)
*B. zhangzhouensis* DW5-4^T^	Aquaculture water of shrimp farm	Contigs	106	3.74	41.40	JOTP00000000.1	Liu et al. (2016)
*B. altitudinis* 41KF2b^T^	Cryogenic tubes for air-sampling at high altitudes	Contigs	39	3.68	41.30	ASJC00000000.1	Lai et al. (2014a)
*B. australimaris* NH7I_1^T^	Surface sediment of shrimp farm	Scaffolds	31	3.65	41.30	LGYN00000000.1	Liu et al. (2016)
*B. xiamenensis* HYC-10^T^	Intestinal contents of flathead gray mullet (*Mugil cephalus*)	Contigs	134	3.61	41.30	AMSH00000000.1	Lai et al. (2012)
*B. pumilus* 145	Shallow water system	Complete^2^	1	4.05	41.16	CP027116.1	Zarza et al. (2018)
*B. pumilus* 150a	Shallow water system	Complete	1	3.74	41.40	CP027034.1	Zarza et al. (2018)
*B. pumilus* PDSLzg-1	Oil-contaminated soil	Complete^2^	1	3.71	41.99	CP016784.1	Hao et al. (2016)
*B. pumilus* PE09-72	Sponge	Contigs	55	3.69	41.20	MIJA00000000.1	Matobole et al. (2017)
*B. pumilus* RI06-95	Pettaquamscutt River	Scaffolds	16	3.64	41.60	LFGZ00000000.1	Hamblin et al. (2015)
*B. pumilus* Ha06YP001	Commensal bacteria from the American Lobster (*Homarus americanus*)	Scaffolds	13	3.64	41.60	PTXV00000000.1	Ranson et al. (2018)
*B. pumilus* SF14	Seawater	Contigs	18	3.63	41.70	LHCE00000000.1	Di Luccia et al. (2015)
*B. subtilis* subsp. *subtilis* 168	Mutagenic strain from *B. subtilis* Marbug	Complete	1	4.21	43.5	NC_000964.3	Borriss et al. (2018)

*^1^Classification in accordance with the NCBI Genome Database. ^2^Chromosome data (plasmid or other smaller unknown sequences also available); ^*T*^, Type strain.*

Among seven different housekeeping genes, Liu et al. (2013) highlighted the gyrase B-coding gene as a valuable alternative for the assessment of the phylogenetic diversity of bacteria in the *B. pumilus* clade, when analyzing marine and terrestrial-derived strains. In this sense, it is important to use at least another reliable marker gene for accurate phylogenetic inference, particularly for the *B. pumilus* clade, whose taxonomy has been extensively restructured within the last few years (Branquinho et al., 2015; Liu et al., 2015, 2016). Owing to this confirmed higher resolution of the *gyrB* housekeeping gene in distinguishing marine strains from the *B. pumilus* group (Liu et al., 2013), we performed the phylogenetic analyses with the *gyrB* sequences from the members of this clade and the marine-derived *B. pumilus* strains: *B. pumilus* ATCC 7061^T^ (ABRX01000004.1), *B. safensis* F0-036b (AY167867.1), *B. zhangzhouensis* DW5-4^T^ (JOTP01000022.1), *B. altitudinis* 41KF2b^T^ (ASJC01000015.1), *B. australimaris* NH7I 1 (LGYN01000023.1), *B. xiamenensis* HYC-10^T^ (AMSH01000062.1), *B. pumilus* 145 (CP027116.1), *B. pumilus* 150a (CP027034.1), *B. pumilus* PDSLzg-1 (CP016784.1), *B. pumilus* PE09-72 (NZ MIJA01000032.1), *B. pumilus* RI06-95 (NZ_LFGZ01000003.1), *B. pumilus* Ha06YP001 (NZ PTXV01000002.1), and *B. pumilus* SF214 (LHCE01000018.1).

These sequences were all aligned with the ClustalW tool within the interface of the MEGAX software (Kumar et al., 2018). The neighbor-joining (NJ) method (Saitou and Nei, 1987) was selected for the 16S rRNA coding sequence and the maximum likelihood (ML) method (Felsenstein, 1981) for the *gyrB* marker. Following the best-fit substitution model, NJ and ML trees were constructed using Tamura-Nei (Tamura and Nei, 1993) and Kimura-2 (Kimura, 1980) models, respectively. Tree topologies were guaranteed by the bootstrap test with 1000 (NJ) and 100 replicates (ML). Four 16S rRNA gene sequences from different microbial strains were used as outgroups: *Bacillus subtilis* subsp. *subtilis* 168 (NC 000964.3) as a genus relative, *Clostridium oceanicum* DSM 1290T (FR749925.1) as a Firmicutes counterpart, *Ruegeria pomeroyi* DSS-3 (NR_028727.1) as a Gram-negative representative and *Thermococcus litoralis* JCM 8560 (AB603515.1) as the ultimate outgroup. The *gyrB* gene sequences from *B. subtilis* subsp. *subtilis* 168 (NC_000964.3) and *Haloferax mediterranei* ATCC 33500 (AOLO01000002.1) were selected as outgroups. Finally, the trees were depicted with FigTree^1^.

### Genome-Based Taxonomy

The overall genome-related index (OGRIs) (Chun and Rainey, 2014) was calculated to evaluate potential relatedness of the sponge-associated *B. pumilus* 64-1 strain with those from the *B. pumilus* group. Seven genomes of aquatic-derived *B. pumilus* isolates deposited in the GenBank database^2^ were incorporated into this analysis. The complete genome sequence of the *B. subtilis* 168 strain consisted of the external group. A summary of basic genomic aspects of the elected *Bacillus* type strains can be found in Table 1.

Average nucleotide identity (ANI) values were determined by the ANI calculator, part of the enveomics toolbox (Rodriguez-R and Konstantinidis, 2016) and the JSpeciesWS (Richter et al., 2016). The identity thresholds of ≥95% for ANIb (estimated by the BLAST algorithm) and ≥96% for ANIm (calculated by the MUMmer algorithm) were adopted for species delineation (Rosselló-Móra and Amann, 2015). A Pearson correlation matrix of the ANIm values (JSpeciesWS) was built and finally plotted hierarchically with the R corrplot package (Wei and Simko, 2017).

JSpeciesWS was also employed for estimation of correlation indexes of tetra-nucleotide signatures, in which only regression values above 0.999 were taken in consideration to group strains in the same species (Rosselló-Móra and Amann, 2015). The Genome-to-Genome Distance Calculator (GGDC 2.1) was applied to the assessment of the digital DNA-DNA hybridization (dDDH), assuming a 70% cutoff (Meier-Kolthoff et al., 2013) and a maximum degree of 1.0% for variation in the genomic G + C content within the same species (Meier-Kolthoff et al., 2014). The Microbial Genome Atlas (MiGA) webserver (Rodriguez-R et al., 2018) was also employed for taxonomic classification of the *B. pumilus* 64-1 genome against the updated databases of reference genome sequences (NCBI Prok) and type strains genomes (TypeMat). The BLAST Ring Image Generation (BRIG) program was applied to depict comparisons between the *B. pumilus* 64-1 genome with those strains for which the highest OGRIs was obtained (Alikhan et al., 2011).

### Core and Pangenome Analyses

The pangenome of this group of strains (*B. pumilus* 64-1, aquatic-derived *B. pumilus*, and *B. pumilus* ATCC 7061^T^) was estimated by a CDS multiclustering approach from the GET_HOMOLOGUES package (Contreras-Moreira and Vinuesa, 2013), using a 75% of pairwise alignment coverage and e-value set at 10^−5^. The core genome was calculated with all three clustering algorithms: bidirectional best hit (BDBH), COGtriangles and OrthoMCL, the pangenome with the COGtriangles and OrthoMCL algorithms. The Venn diagrams for the clustered orthologous genes were plotted with the InteractiVenn tool (Heberle et al., 2015). The pangenome compartments, “core,” “soft core,” “cloud,” and “shell,” were obtained with the GET_HOMOLOGUES script “parse pangenome matrix.pl.” Briefly, the “core” comprises the genes common to all the analyzed genomes; the “soft-core,” all the genes found in 95% of the genomes, the “cloud” component only the genes present in a minority of genomes and the “shell,” the rest of the genes found in several genomes (Contreras-Moreira and Vinuesa, 2013). The theoretical sizes of the core- and pan-genomes were estimated with OrthoMCL derived gene families, applying both the Tetellin and Willenbrock exponential models. To exclude any bias due to the differences between the original programs used to annotate these *B. pumilus* genomes, they were priory reannotated with the DFAST software, the same employed for the structural annotation of the *B. pumilus* 64-1 strain.

### Nucleotide Sequence Accession Number

The draft genome sequence of *B. pumilus* 64-1 strain was submitted to the NCBI GenBank database under the accession number NZ_VSRW00000000.1 and the version described in this paper is the first version.

## Results and Discussion

### Genome Properties

The *B. pumilus* genome was assembled into 16 contigs with an N_50_ of 92,464 bp and coverage of 190×. The final assembly had a very high level of completeness (99.59%) with no contamination following evaluation with the CheckM tool. The genome has approximately 3.66 Mbp with a 41.5% GC content. A total of 3,705 CDS, five rRNA and 45 tRNA-coding genes were assigned, with an estimated coding ratio of 88.3% (Supplementary Table 1). These results were comparable with data derived from other *B. pumilus* genomes deposited in the NCBI GenBank database, for which the average values comprise a genome size of 3.83 Mbp, with a GC content of 41.3% and around 3,784 protein-coding genes. Plasmid sequences were not found using the PlasmidFinder 2.0 tool. Plasmid DNA extraction further confirmed the absence of plasmids in the *B. pumilus* 64-1 strain (data not shown).

From the 3,589 CDS scanned in the eggNOG database, a total of 94.12% could be assigned to one of the functional COG classes. Even though the majority were classified to the Function unknown (“S”) category (Table 2). A considerable number of genes within this category had at least a broad function attributed to them, such as cellular localization or even an enzyme class. Excluding the Function unknown (“S”) class, the other common categories were Transcription (“K”), Amino acid transport and metabolism (“E”) and Cell wall/membrane/envelope biogenesis (“M”).

**TABLE 2:** Functional annotation of orthological groups.

Code	Value	% of 3,082	Description
D	45	1.46	Cell cycle control, cell division, chromosome partitioning
M	167	5.42	Cell wall/membrane/envelope biogenesis
N	37	1.2	Cell motility
O	80	2.6	Post-translational modification, protein turnover, and chaperones
T	97	3.14	Signal transduction mechanisms
U	27	0.88	Intracellular trafficking, secretion, and vesicular transport
V	53	1.72	Defense mechanisms
J	181	5.88	Translation, ribosomal structure, and biogenesis
K	296	9.6	Transcription
L	126	4.09	Replication, recombination, and repair
C	156	5.06	Energy production and conversion
E	253	8.21	Amino acid transport and metabolism
F	89	2.89	Nucleotide transport and metabolism
G	175	5.67	Carbohydrate transport and metabolism
H	102	3.31	Coenzyme transport and metabolism
I	90	2.92	Lipid transport and metabolism
P	170	5.51	Inorganic ion transport and metabolism
Q	46	1.5	Secondary metabolites biosynthesis, transport, and catabolism
S	892	28.94	Unknown

*Genes associated with more of one class (296) were excluded from the number exclusively associated with one class showed in this table.*

### Phylogenetic and Genome-Based Taxonomy Analyses

**FIGURE 1:**
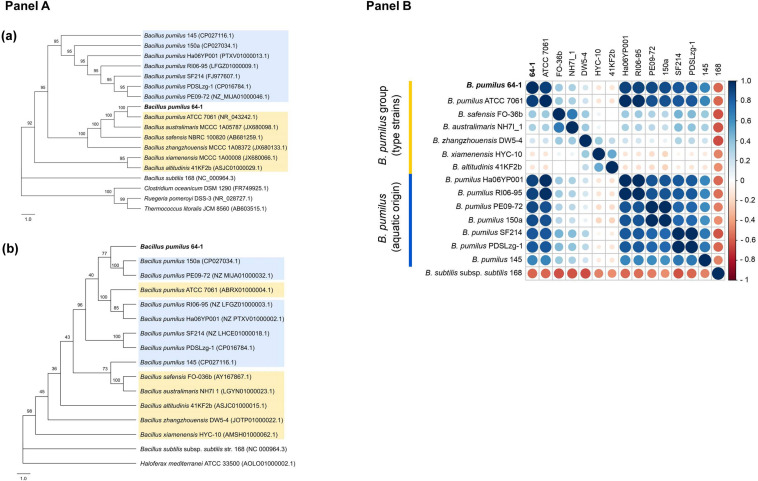
**(Panel A)**: Phylogenetic trees with the *B. pumilus* 64-1 with selected representatives (*B. pumilus* clade shaded in yellow and aquatic-derived *B. pumilus* shaded in blue) based on (a) its 16S rRNA and (b) *gyrB* gene sequences, using the NJ and ML methods, respectively. Bootstrap values are shown at branch nodes. Bar, 1.0 nucleotide substitution (Kunits) rates. For the 16S rRNA NJ tree, *B. subtilis* subsp. *subtilis* 168 (NC 000964.3), *C. oceanicum* DSM 1290T (FR749925.1), *R. pomeroyi* DSS-3 (NR 028727.1) and *T. litoralis* JCM 8560 (AB603515.1) were chosen as outgroups. For the *gyrB* ML tree, *B. subtilis* subsp. *subtilis* 168 (NC 000964.3) and *H. mediterranei* ATCC 33500 (AOLO01000002.1) were set as outgroups. Trees were generated by MEGAX software and depicted with FigTree. **(Panel B)**: Correlation plot based on ANIm values (%) obtained by the JSpeciesWS. The R program was used to the construction of a Pearson correlation matrix using the ANIm values derived from the JSpeciesWS and, posteriorly, for it’s plotting with the corrplot package. The size of the circles is proportional to the ANIm identity value (%). On the left, the key color (red to blue) refers to the increasing level of ANI identity between the pair of isolates.

The 16S rRNA phylogeny reconstruction resulted in the confirmation of a closer association of the *B. pumilus* 64-1 strain with *B. pumilus* ATCC 7061^T^, while the aquatic-derived *B. pumilus* were present onanother major tree branch (Figure 1, Panel Aa). The analysis was replicated using different evolutionary models and the same clade arrangement was verified for the NJ trees. The outcome was somewhat different using the *gyrB* marker gene: *B. pumilus* 64-1 clustered together with the *B. pumilus* 150a and *B. pumilus* PE09-72 (Figure 1, Panel Ab). All *B. pumilus* strains are also distinctively grouped when compared with the other species of the *B. pumilus* clade in the *gyrB* tree (Figure 1, Panel Ab).

To provide a clearer picture of the taxonomic status of *B. pumilus* 64-1 and its closely selected relatives and, in particular, a possible genomic identity of the hitherto described aquatic-derived *B. pumilus*, several genomes alignment-dependent (ANIm, ANIb, and dDDH) and alignment-independent (tetranucleotide composition and GC content) methods were employed. We also used the an OGRI-based genome classifier (MiGA) to ascertain the species affiliation of strain 64-1 and its relatedness with other *B. pumilus* and *Bacillus* spp.

In agreement with the molecular identification, the isolate 64-1 was confirmed to belong to the *B. pumilus* species. Sharing ANIm and ANIb values above the accepted ≥95–95 – 96% threshold for species delineation (Rosselló-Móra and Amann, 2015), the *P. cyanorosea*-isolated strain has as the closest genome relatives the aquatic-derived *B. pumilus* RI06-95 and *B. pumilus* Ha06YP001, the reference strain *B. pumilus* ATCC 7061^T^, followed by two other aquatic strains: *B. pumilus* 150a and *B. pumilus* PE09-72 (Figure 1, Panel B). Accordingly, the tetranucleotide regression values and the dDDH percentages were also higher for *B. pumilus* RI06-95 and *B. pumilus* Ha06YP001. The other species from the *B. pumilus* clade had the lowest calculated metrics (Supplementary Table 2).

Apart from the type strain *B. pumilus* ATCC 7061^T^, the genomic metrics point out that the *B. pumilus* 64-1 isolate clustered together with aquatic-derived *B. pumilus* strains (Supplementary Table 2), none of which had been recovered from sponge samples. The first one, *B. pumilus* RI06-95, is from a freshwater environment and with relevant probiotic potential in shellfish aquaculture systems (Hamblin et al., 2015), while the second closest relative is *B. pumilus* Ha06YP001, a marine bacterium isolated from an American lobster (Ranson et al., 2018). The *B. pumilus* 150a, isolated from a shallow-water system (Zarza et al., 2018), and the *B. pumilus* PE09-72, a symbiont from a marine demosponge (Matobole et al., 2017), clustered together with the *P. cyanorosea*-derived strain in the *gyrB* phylogenetic inference. In summary, genome-based analyses have corroborated the taxonomy of strain 64-1 as belonging to the *B. pumilus* species and guided us to select these aquatic-derived *B. pumilus* isolates, together with the *B. pumilus* ATCC 7061^T^, for further short-scale pangenomic assessment.

### Core and Pangenome Analyses

The orthologous clustering of 33,938 CDS from the nine genomes resulted in 5,592 consensus gene clusters, the calculated pangenome (Supplementary Figure 2A). Of these, 2,984 clusters (53.36%) were found in all the studied bacilli, constituting the core genome (Supplementary Figure 2B). The computation of the flexible genome corroborated this result, with the “cloud” compartment summing up the genes found on two or fewer genomes, the “shell” as the complement of genes present on seven genomes; and the “soft core” refereeing to those encountered on eight genomes (Supplementary Figure 3).

While the relatively high proportion of the core genome might be explained by the analyses being conducted with a restricted number of strains from the same species, subsequent analyses indicate that the pangenome of these *B. pumilus* strains is open (Supplementary Figures 2C,D). Recent pangenome analyses for the other major *Bacillus* clades – *B. subtilis* (Wu et al., 2020), *B. cereus* (Bazinet, 2017), and *B. amyloliquefaciens* (Chun et al., 2018) – also indicate the presence of open pangenomes with a smaller set of the core genetic repertoire due to the increased number of genomes analyzed, endorsing the huge and widely known metabolic diversity in these ubiquitary *Bacillus* groups.

Analyzing the *B. pumilus* specific genes, none of them were associated with survival in the marine and sponge niches. In fact, most of these strain specific genes were either annotated as hypothetical proteins, assigned to the Function unknown (“S”) class or with no function attributed during the eggNOG functional characterization; or consisted of a prophage or transposable-related gene (data not shown). Therefore, with the exception of particular mobilome components, the further addressed potential ecological and biotechnological relevant genes seem to not be part of the accessory component of the *B. pumilus* 64-1 genome.

### Prediction of Secondary Metabolite Gene Clusters

Twelve BGCs were identified in the *B. pumilus* 64-1 strain by the antiSMASH software. These included clusters potentially encoding for five non-ribosomal peptide synthetases (NRPS), one type III polyketide synthase (PKS), one hybrid NRPS-T1PKS; one bacteriocin, two betalactones and two terpenes. One BGC had high homology with the bacilysin gene clusters in *B. velezensis* FZB42 (BGC0001184.1) and *Bacillus* sp. CS93 (BGC0000888.1), displaying 85 and 80% similarity, respectively, (Table 3 and Supplementary Figure 4A). Bacilysin is a non-ribosomally synthesized dipeptide composed of an N-terminal L-alanine residue and a C-terminal L-anticapsin residue. Released upon the action of peptidases, it acts as a competitive inhibitor of glucosamine-6-phosphate synthetase, affecting the supply of a key monomeric precursor of bacterial and fungal cell walls, which leads to cell lysis and explains its activity against a wide range of microbial strains (Wang et al., 2018).

**TABLE 3:** BGCs clusters identified in the *B. pumilus* 64-1 genome by the antiSMASH platform.

Cluster	Type	Most similar known cluster	% Similarity
1	Bacteriocin	–	–
2	Other/NRPS	Bacilysin	85%
3	NRPS	Bacilibactin	53%
4	Terpene; siderophore	Carotenoid; *iucA* and *iucC* (iron transporter)	50%
5	Betalactone	Fengycin	53%
6	Betalactone	–	–
7	T3-PKS	–	–
8	Terpene	–	–
9	NRPS-T1PKS hybrid	Paenilamicin	35%
10	NRPS	Lichenysin	50%
11	NRPS	Surfactin	39%
12	NRPS	Surfactin	8%

*–, No reference value; NRPS, non-ribosomal peptide synthetase; PKS, polyketides synthase.*

Bacilysin is encoded by the *bacABCDE* operon and its synthesis involves at least six different enzymatic reactions starting with a prephenate intermediate in all *Bacillus* species in which this BGC has been detected (Steinborn et al., 2005). Each enzyme is encoded for by its respective gene within the core cluster, apart from the *bacE* gene, which has been categorized as an L-amino acid ligase participating in the export of bacilysin. Additionally, the *bacF* gene encodes the transaminase BacF which is involved in the penultimate step in the formation of the dipeptide, and the *bacG* gene, encoding the NADPH-dependent oxidoreductase BacG, which provides the epoxide substrate for the BacF enzyme (Özcengiz and Öğülür, 2015). Apart from the *bacE* gene, all the *bacABCDE* operon genes were present as a cluster in the *B. pumilus* 64-1 genome (Supplementary Figure 4A). As previously mentioned, the cluster displayed a high level of identity with the bacilysin gene clusters in *B. velezensis* FZB42 and *Bacillus* sp. CS93. The only differences between the bacilysin gene cluster in *B. pumilus* 64-1 are the presence of the *bacE* gene in the *B. velezensis* FZB42 *bac* operon and the absence of the *bacA* gene in the *Bacillus* sp. CS93 *bac* operon. There is the possibility that the *bacE* gene may be located in another region of the *B. pumilus* 64-1 genome, typically mediated by *bacE* in other *Bacillus* strains such as *B*. *velezensis*, or that the role in bacilysin transport is being undertaken by the product of another as yet unidentified gene. A *B. zhangzhouensis* PE09-72, isolated from the Demospongiae *Isodictya compressa* collected in Algoa Bay, harbored at least eight secondary metabolic pathways included three NRPS, of which one PKS/NRPS hybrid, one terpene and two bacteriocins namely, bacilysin and lichenysin. Interestingly similar to the *B. pumilus* 64-1 strain, the *bacE* gene was also not present within the *bacABCDE* operon in the *B. zhangzhouensis* PE09-72 genome (Matobole et al., 2017).

Following analysis using the BAGEL 4 software, two contigs encoding genes potentially related to the biosynthesis of amylocyclicin, sactipeptides and the bacteriocin UviB precursor were identified in our strain (Supplementary Figure 4B). Analyzing the plant-associated *B. amyloliquefaciens* FZB42, Scholz et al. (2014) elucidated a cluster of six genes responsible for the metabolism and export of amylocyclicin, which is a ribosomally synthesized class II bacteriocin with strong inhibitory activity against several Gram-positive bacteria. The UviB protein was first described in a *Clostridium perfringens* plasmid pIP404, encoded by a transcriptional unit, *uviABC*, whose function was related to assisting in the secretion of the UV-inducible bacteriocin BCN5 (Gamier and Cole, 1988). However, the isolated presence of a single UviB-like protein may not correspond to a functionally operative *uviABC* operon. But is important to emphasize that the *uviB* gene identified by the BAGEL4 software encodes a membrane-disrupting holin, previously described in a *B. pumilus* strain and which shares high similarity to the bacteriocin UviB of *C. perfringens* (Aunpad and Panbangred, 2012). The sporulation killing factor (SKF) is one of the three types of sactipeptides already identified in *Bacillus* spp., including in *B. pumilus*, such as identified in the strain 64-1 by BAGEL4 in the present study. In high concentrations, this peptide has been proved to inhibit the growth of other bacteria (Zhao and Kuipers, 2016).

Together with metabolomics data, “genome scanning” applied to aquatic or marine-derived *B. pumilus* has proven useful in unveiling a number of BGCs that encode for antimicrobial metabolites, such as amicoumacin (Hamblin et al., 2015), pumilacidin (Saggese et al., 2018), amides (Zhou et al., 2018), and bacteriocins (Zarza et al., 2018). Our results reinforce the potential that *B. pumilus* 64-1 strain may possess the capacity to produce a range of different bioactive peptides, which may find utility in the treatment of MDR microbial infections (Freitas-Silva et al., 2020), particularly given that sponge-associated strains of *B. pumilus*, including *B. pumilus* 64-1, have previously been reported to have activity against MDR strains of *Escherichia coli, Citrobacter freundii, K. pneumoniae, Neisseria gonorrhoeae, Staphylococcus* spp., *Streptococcus* spp., and vancomycin-resistant *Enterococcus* (VRE) (Santos et al., 2010; Freitas-Silva et al., 2020).

### Screening for Genes Encoding Carbohydrate-Active Enzymes (CAZymes) and the Proteolytic Repertoire

In total, 77 genes potentially encoding CAZymes – mostly from the glycoside hydrolase (GH) and glycosyltransferase (GT), followed by carbohydrate esterase (CE) and polysaccharide lyase (PL) classes – were identified in the *B. pumilus* 64-1 genome (Supplementary Figure 5A). Apart from the presence of GHs potentially involved in starch (GH126 and GH13) and cellulose breakdown (GH9 and GH48), it is noteworthy that complete sets of genes were present for the degradation of pectin (GH28, GH105, PL1, PL6, and CE8), chitin (GH18, CE4, CE6, and CE14) and xylan (GH30, GH43, GH51, GH10, GH11, CE1, and CE7) (Supplementary Table 3). Interestingly, we have found potential xylanase-coding genes (GH10 and GH11 families), some of them co-located with the cellulolytic GH genes (Supplementary Figure 5B), indicating the likelihood of their potential coordinated co-expression for the breakdown of plant-derived polysaccharides. Novel xylanases have been reported from some marine-derived *Bacillus* spp. for a range of applications including the enzymatic hydrolysis of seaweed biomass (Parab et al., 2017) and the production of xylobiose from agricultural residues (Khandeparker et al., 2017). Given the broad applicability of these enzymes, further heterologous expression of the GH10 and GH11 (endo-1,4-β-xylanases) genes and their subsequent biochemical characterization from this sponge-derived *B. pumilus* 64-1 strain may prove fruitful.

Other genes possibly linked to xylan metabolism were also identified including a sialic acid-specific acetylesterase from the carboxyethylesterase family 6 (*axeA*), which appears to contain specific domains for the esterification of this acidic sugar. Sialidases and sialic-acid modifying enzymes have been characterized as important effectors for evasion of the human host immune response in bacterial pathogens (Severi et al., 2007) or in assisting members of the human microbiome in establishing on the intestinal mucosa (Vimr, 2013). Sialylated molecules are nevertheless likely to be present in the sponge mesohyl (Müller et al., 1976), and genomes of other sponge microbial symbionts harbored putative sialidases, with the suggestion of this being linked to their capacity to degrade these sialylated molecules in the sponge extracellular matrix (Kamke et al., 2013; Bayer et al., 2018; Podell et al., 2019).

In addition, approximately 6.5% of the CDS were affiliated to various peptidase families and/or to peptidase inhibitors, with the majority being associated with some essential metabolic process in the *B. pumilus* species. In particular, we identified an intracellular collagenase from the ample peptidase U32 family (MER0123646). The majority of U32 collagenolytic proteases are known to function as virulence factors in human pathogenic bacteria (Zhang et al., 2015). Furthermore, the collagenase annotated from *B. pumilus* 64-1 shares 35.4% similarity with a spongin-degrading collagenase from the *Pseudoalteromonas agarovirans* NW4327, a primary pathogen of the Great Barrier Reef sponge *Rhopaloeides odorabile* (Mukherjee et al., 2009). Further work will be required to determine whether *B. pumilus* 64-1 does possess *in vitro* collagenolytic activity to subsequently assign a role to this enzyme in the association of *B. pumilus* 64-1 with the *P. cyanorosea* host.

### Genomic Features Potentially Associated With Survival in the Marine Environment

For bacteria to survive in marine environments at salinity levels of approximately 3.5%, they must be able to overcome stresses due to both high Na^+^ concentrations as well as high osmotic pressure. They typically respond to variations in external osmotic pressure by relying upon diverse and integrated membrane transport systems and the biosynthesis and accumulation of certain compatible solutes (Yaakop et al., 2016). In this context, the *B. pumilus* 64-1 genome has a number of genes potentially associated with marine survival directly related to osmoregulation and/or homeostasis of intracellular salinity level (data not shown). These genes include those encoding for: (i) mechanosensitive channels from the MscS (*yhdY*, *ykuT*, and *yfkC*) and MscL families (*mscL*); (ii) an antiporter complex involved in resistance to increased cation concentration and alkali conditions (*phaA*, *mrpB*, *phaC*, *mrpD*, *mrpE*, *phaF*, and *phaG*); (iii) components of the potassium importer KtrA (*ktrA* and *ktrB*); and (iv) CrcB fluoride exporters (*crcB*). Control of the biosynthesis of cellular L-proline and glycine betaine pools appears to be important in the *B. pumilus* 64-1 strain given the presence of genes for: (i) the OpuA, OpuB, and OpuC glycine betaine transporters (*opuAC* and *opuAB*); (ii) the osmoprotectant ABC-type proline betaine transport system (*proV*, *opuCD*, *opuCC*, *opuCB*, *opuCA*, *proWX*, and *proV*); (iii) the glycerol uptake facilitator protein (*glpF*); (iv) and enzymes for glycine betaine (*gbsA* and *gbsB*) and proline metabolism (*proA*, *proB*, *proC*, and *ycgM*) (data not shown).

Mechanosensitive channels are recognized as having a role in protecting bacterial cells from the effects of sudden hypotonic shocks (Booth, 2014). The *B. pumilus* 64-1 genome appears to possess at least three different copies of genes potentially encoding for members of the MscS family in addition to one set of genes encoding an MscL channel. Both sets of these families have previously been reported to play important roles in a protective effect against osmotic shock (Booth and Blount, 2012). These mechanosensitive channels, together with Ktr components, the Opu glycine betaine uptake systems and the fine cytoplasmic control of glycine betaine and proline levels are fundamental to the “salt out” tactics employed by *B. subtilis* (Hoffmann and Bremer, 2017) and in *B. licheniformis* (Schroeter et al., 2013). A similar set of protective actions has not to date been comprehensively described for any member of the *B. pumilus* group. To our knowledge, the only report where a terrestrial and marine *Bacillus* strains were compared in terms of survival under hyperosmolarity was 20 years ago: the genome of an alkalophilic *Bacillus halodurans* strain was enriched in ABC transport systems when compared to a soil-born *B. subtilis* (Takami et al., 2000).

### Identification of Antimicrobial Resistance and Metal Tolerance Genes

The *cat-86* gene, encoding a chloramphenicol acetyltransferase (CAT), was identified along with an extended-spectrum BPU (from *B. pumilus*) β-lactamase, an identity level superior to 90% as expressed by the RGI tool. However, the functional annotation reveals the theoretical capacity of the *B. pumilus* 64-1 strain to be resistant to representatives of other antimicrobial classes, including chloramphenicol, aminoglycosides, fosfomycin, tetraciclins, cyclic peptides, glycopeptides and betalactams (Supplementary Table 4). No resistance phenotype was however observed after disk diffusion susceptibility tests with the strain 64-1 (data not shown); although, this may mean that the genotype was not expressed under the tested conditions. Future application of more discriminatory antimicrobial susceptibility tests, such as microdilution-based methods using increasing concentrations of antimicrobials from each representative class, would confirm this resistance profile.

Genes for transcriptional regulators involved with antibiotic resistance, such as TetR and MarR, and for the response regulator OmpR, were also identified in the *B. pumilus* 64-1 genome (Supplementary Table 4), showing that the *B. pumilus* 64-1 strain may be able to control the expression of these genes under the conditions imposed by these antagonist compounds. Moreover, the functional annotation of multidrug resistance proteins (*ykkC*, *ykkD*, *ebrA*, *ebrB*, *mdtI*, and *sugE*) and multidrug efflux pumps (*yhbJ* and *norM*), including those from the multidrug and toxic compound extrusion (MATE) family (*yisQ* and *yoeA*) and the RND (resistance-nodulation-cell division) drug exporters (*ydgH*, *mdtC*, and *swrC*), suggest a wider intrinsic resistance, as verified for the *B. cereus* group, either for their chloramphenicol and tetracycline genetic repertoire (Glenwright et al., 2017) or by the conservation of these small molecules efflux pumps (Hassan et al., 2017). This apparent intrinsic resistance genotype of the strain 64-1 may not only form part of defense strategies against the ever-changing environmental conditions but also for physiological processes, considering that some of the substrates for these multidrug transporters are cations, such as Na^+^ and H^+^ (Du et al., 2018).

The *B. pumilus* 64-1 also appears to possess an extensive genetic reservoir for potential tolerance to several heavy metals, comprising arsenic, mercury, tellurium, cobalt, cooper and chromate (Supplementary Table 4). Likely arsenic resistance is particularly evident, with the presence of an *ars* operon, including all the genetic elements required for cellular detoxification of the metal. The presence of these genes in *B. pumilus* 64-1 is perhaps not surprising given that arsenic tolerance has previously been reported in *B. pumilus* strains isolated from various environments (Raja and Omine, 2012; Khowal et al., 2017; Nithya et al., 2017; Titah et al., 2018; Dolphen and Thiravetyan, 2019). Additionally, marine sponge-associated *Streptomyces* strains have also previously been reported to be enriched in arsenic transport genes (Jackson et al., 2018) and the sponge-associated *Entotheonella* spp. are known to sequester arsenic in intracellular vesicles whilst residing in their sponge host, *Theonella swinhoei* (Keren et al., 2017). Future work on the potential degradation of arsenic by the *B. pumilus* 64-1 strain might clarify its applicability for bioremediation or biosorption processes in marine environments.

### CRISPR-Cas System and Mobilome Elements

CRISPR–Cas systems are known to be part of the molecular armamentarium employed by sponge microbial symbionts against viral attack. Ultimately, these sequences can influence the evolution of their genomes (Horn et al., 2016; Alex and Antunes, 2018; Burgsdorf et al., 2019). A functional class 1 CRISPR was detected on the *B. pumilus* 64-1 genome, being classified as a type III-B CRISPR-Cas10 (Couvin et al., 2018). This region harbors the type III-signature protein, the ssDNase Cas10, and the five spacers, coding for the Cmr1, Cmr3, Cmr4, Cmr5, and Cmr6 proteins, with conservation DNA identities of 86.14% for the DR and 7.14% for the spacers. The Cmr5 protein was confirmed during the annotation step (Supplementary Figures 6A,B). One spacer showed similarity with spacers from the genome of *B. safensis* KCTC 12796BP, a strain which was also isolated from a marine sponge in the Jeju Island, South Korea (Hanh et al., 2018). Type III-B systems shows *in vivo* specificity toward the mRNA of invasive virus and foreign DNA by an RNA-targeted mode (Tamulaitis et al., 2017). Therefore, there is the likelihood that *B. pumilus* 64-1 may use this CRISPR as an additional defense system against phages and/or other mobile elements.

No entire genomic island (GI) was identified in the *B. pumilus* 64-1 genome. Four incomplete regions for temperate prophages were detected by the PHASTER server; one assigned to the family Myoviridae, *Brevibacillus* phage Jimmer1, and the other three to the family Siphoviridae: *Bacillus* phage phi105, *Bacillus* phage Wbeta, *Bacillus* phage G (data not shown). Using the ISfinder database, BLASTx searches produced significant alignments with IS*1182*-like elements, all originally characterized in Bacillaceae, including the IS*Bpu1*, specific for the *B. pumilus* species (data not shown). All these IS harbor one single ORF for a transposase (Siguier et al., 2015) and were previously shown to mediate the transfer of antimicrobial resistance and toxin genes in clinically important Firmicutes pathogens (Mikalsen et al., 2015; Smith et al., 2015; Furi et al., 2016). A manual inspection of the eggNOG annotation files allowed the identification of 76 prophage genes (Supplementary Table 5), some of them matching exactly to the segments confirmed after scanning by PHASTER program. Genes for an IS66 transposase and three putative transposases were also observed during the curation of the functional annotation (Supplementary Table 5).

*Bacillus pumilus* was found to have a percentage of horizontally transferred genes (%HGT) of around 2.16% which is similar to the 2.52% for the type-strain *B. pumilus* ATCC 7061^T^ (IMG Genome ID: 642791616), and higher than all the twenty *B. pumilus* genomes with calculated %HGT upon comparison with the [Integrated Microbial Genomes and Microbiomes (IMG/M) Database v. 5.0] (Chen et al., 2019). HGT has been credited to be the additional important component in the cross-regulation of marine animal-bacterial interactions (Degnan, 2014) and may even engage bacteria in symbioses with some eukaryotic hosts (Tong et al., 2020). A higher mobilome content may thus reflect the ability of the *B. pumilus* 64-1 strain to continuously acquire and exchange new and advantageous metabolic functions from the other members constituting the sponge-associated and the environmental microbiomes and, even, the invertebrate host.

## Conclusion

Owing to its previously reported antimicrobial activity, the genome of the sponge-derived *B. pumilus* 64-1 strain was sequenced and subsequently analyzed employing a variety of different bioinformatic tools. The isolate was confirmed to have the type strain of the *B. pumilus* clade (*B. pumilus* ATCC 7061^T^) and other aquatic-derived *B. pumilus* strains (*B. pumilus* RI06-95 and *B. pumilus* Ha06YP001) as its closest relatives, following phylogenetic and genome-based taxonomic analyses. In addition, together with these related strains, it appears to have an open pangenome, with a dominant core component and specific accessory genes distributed throughout these strains.

A number of BGCs were identified one of which potentially encodes for the antimicrobial peptide bacilysin. This gene cluster exhibited high similarity (85%) to the corresponding *bac* operon and may be responsible for the bioactivity previously reported for this isolate. Further genome mining revealed a variety of metabolic traits with interesting ecophysiological and biotechnological significance. The enhanced survival of the strain in the marine habitat is likely to be due to a high number of genes which encode for transporters and osmolytes. Sequences encoding for lignocellulose-degrading CAZymes depicts the potential applicability of this strain for the bioconversion of plant-derived feedstock in biofuels. Future confirmation of complete arsenic metabolism may also shed more light on the prospective use of the strain 64-1 for bioremediation purposes, once all genes for detoxification of this heavy metal were present in its genome.

The first report on the genomic characterization of a microbial isolate from a Homoscleromorpha sponge was for the *Bacillus plakortidis* P203 (Wang et al., 2016), a strain isolated from the marine sponge *Plakortis simplex* (Borchert et al., 2007). This current work is the second to present the genome of a bacterial isolate from another homoscleromorph sponge and the first for the recently described *P. cyanorosea* species (Muricy et al., 2019), and for the *Plakina* genus. Increased information on cultured isolates from different sponge species will shed further light on marine sponge microbiomes (Laport, 2018), considering the growing knowledge on the biology of microbe-sponge interactions (Pita et al., 2018); particularly in often-neglected bacterial taxa studied in this holobiont, namely the *Firmicutes*. As suggested future research, a comparative genomics between *B. pumilus* isolated from sponges, isolated from the aquatic environment, and isolated from the terrestrial environment may prove fruitful. This will allow a further analysis for the enrichment of sponge-host dependent lifestyle genes and marine adaptation in these sponge-derived *Bacillus*. In addition, the bioactive substances produced by *B. pumilus* 64-1 are currently being isolated, purified for further evaluantion of its molecular mechanism of antimicrobial action. All CAZymes activities can also be explored, followed by heterologous expression assays, mainly the ones with degrading abilities in plant biomass.

## Data Availability Statement

The datasets presented in this study can be found in online repositories. The names of the repository/repositories and accession number(s) can be found in the article/Supplementary Material.

## Author Contributions

JF-S and ML conceived the study and designed the experiments. JF-S, GM, and ML performed the experiments. JF-S, BO, and FV analyzed the data. BO, JF-S, AD, and ML wrote the manuscript. All authors contributed to manuscript revision, read and approved the submitted version.

## Conflict of Interest

The authors declare that the research was conducted in the absence of any commercial or financial relationships that could be construed as a potential conflict of interest.

## Footnotes

1 http://tree.bio.ed.ac.uk

2 https://www.ncbi.nlm.nih.gov/genome/genomes/440

## Supplementary Material

Supplementary Figure 1 Workflow of the bioinformatic tools used during the assembly (colored in red) and the annotation (colored in blue) of the *B. pumilus* 64-1 genome. Details about the identification of genes potentially associated, resistome and mobilome elements from the eggNOG functional annotation files are depicted in gray, and black, respectively. ARG, antimicrobial resistance genes; BGCs, biosynthetic gene clusters; CAZymes, carbohydrate-active enzymes; IS, insertion sequences.

Supplementary Figure 2 Estimation of the core and pangenome sizes: Venn diagrams depicting the **(A)** pangenome and **(B)** core genome differentially calculated for the *B. pumilus* 64-1 and the genomic-related *B. pumilus* strains by the GET_HOMOLOGUES software; **(C)** theoretical pangenome size and **(D)** theoretical core genome size applying the Tettellin (blue) and Willenbrock (red) fits by the OrtoMCL algorithm.

Supplementary Figure 3 Barplot with the frequencies of orthologs clusters assigned to core, soft core, shell and cloud categories according to the orthoMCL algorithm by the GET_HOMOLOGUES software.

Supplementary Figure 4 Genomic architecture of the: **(A)** bacilysin gene cluster and its closest BGCs matches (MiBIG accession numbers parenthetical) according to the antiSMASH tool; **(B)** bioactive peptides-encoding genes within the areas of interest (AOIs) detected by the BAGEL4 software.

Supplementary Figure 5 Relevant outcomes about the CAZyme repertoire of the *B. pumilus* 64-1 genome: **(A)** Distribution of CAZymes classes by the dbCAN 2 metaserver. GHs, glycoside hydrolases; GTs, glicosyltransferases; PLs, polysacharides lyases; CEs, carbohydrate esterases. **(B)** Concatenated organization of four putative extracellular cellulases (GH9, GH48, and GH5) and xylanase (GH10) genes.

Supplementary Figure 6 Type III-B CRISPR-Cas10 system identified in the *B. pumilus* 64-1 genome: **(A)** genomic organization adapted by the CRISPRCasFinder program and Artemis. Highlighted in brown the spacer sequence showing similarity with the *B. safensis* KCTC 12796BP genome; **(B)** RNA structure (colored by base-pairing probability) predicted by RNAFolder web server.

Supplementary Table 1 Genome properties of *B. pumilus* 64-1.

Supplementary Table 2 The overall genome-related index (OGRIs) values from different *in silico* bioinformatics tools among *B. pumilus* 64-1 versus members from the *B. pumilus* group and aquatic-derived *B. pumilus* strains.

Supplementary Table 3 List of relevant polyssacharide-degrading CAZymes identified in the *B. pumilus* 64-1 genome.

Supplementary Table 4 List of genes related to antimicrobial resistance and metal tolerance/detoxification annotated in the *B. pumilus* 64-1 draft genome.

Supplementary Table 5 List of mobilome-related elements in the *B. pumilus* 64-1 draft genome detected by the eggNOG-mapper v2.
