# Supplementary figures and images for: Peeling the Layers Away: The Genomic Characterization of Bacillus pumilus 64-1, an Isolate With Antimicrobial Activity From the Marine Sponge Plakina cyanorosea (Porifera, Homoscleromorpha)
Source: Front Microbiol. 2021 Jan 8;11:592735. doi: 10.3389/fmicb.2020.592735 (PMC7820076; doi:10.3389/fmicb.2020.592735)

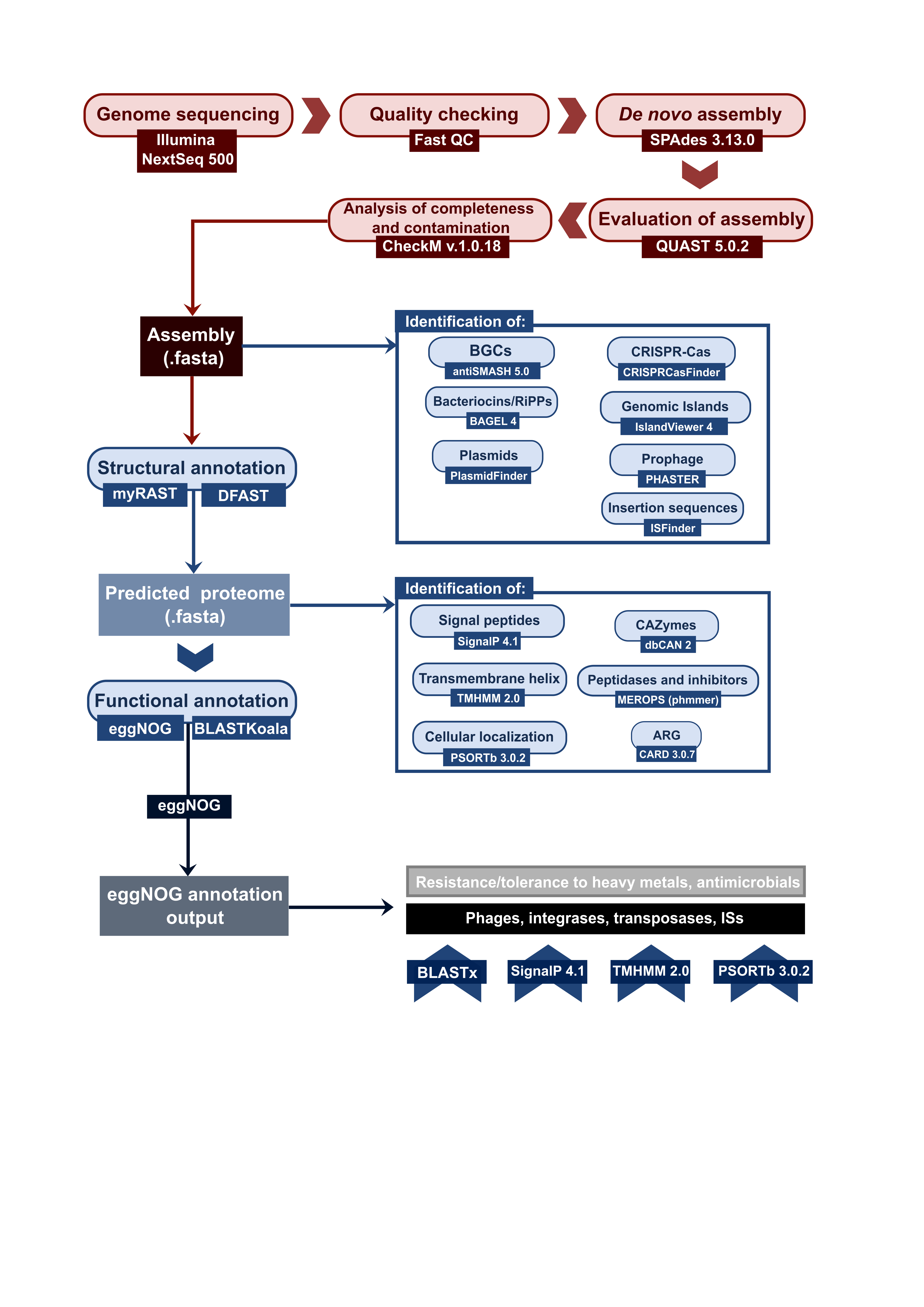

Supplement: Supplementary Figure 1 — Workflow of the bioinformatic tools used during the assembly (colored in red) and the annotation (colored in blue) of the B. pumilus 64-1 genome. Details about the identification of genes potentially associated, resistome and mobilome elements from the eggNOG functional annotation files are depicted in gray, and black, respectively. ARG, antimicrobial resistance genes; BGCs, biosynthetic gene clusters; CAZymes, carbohydrate-active enzymes; IS, insertion sequences. [file Image_1.JPEG]

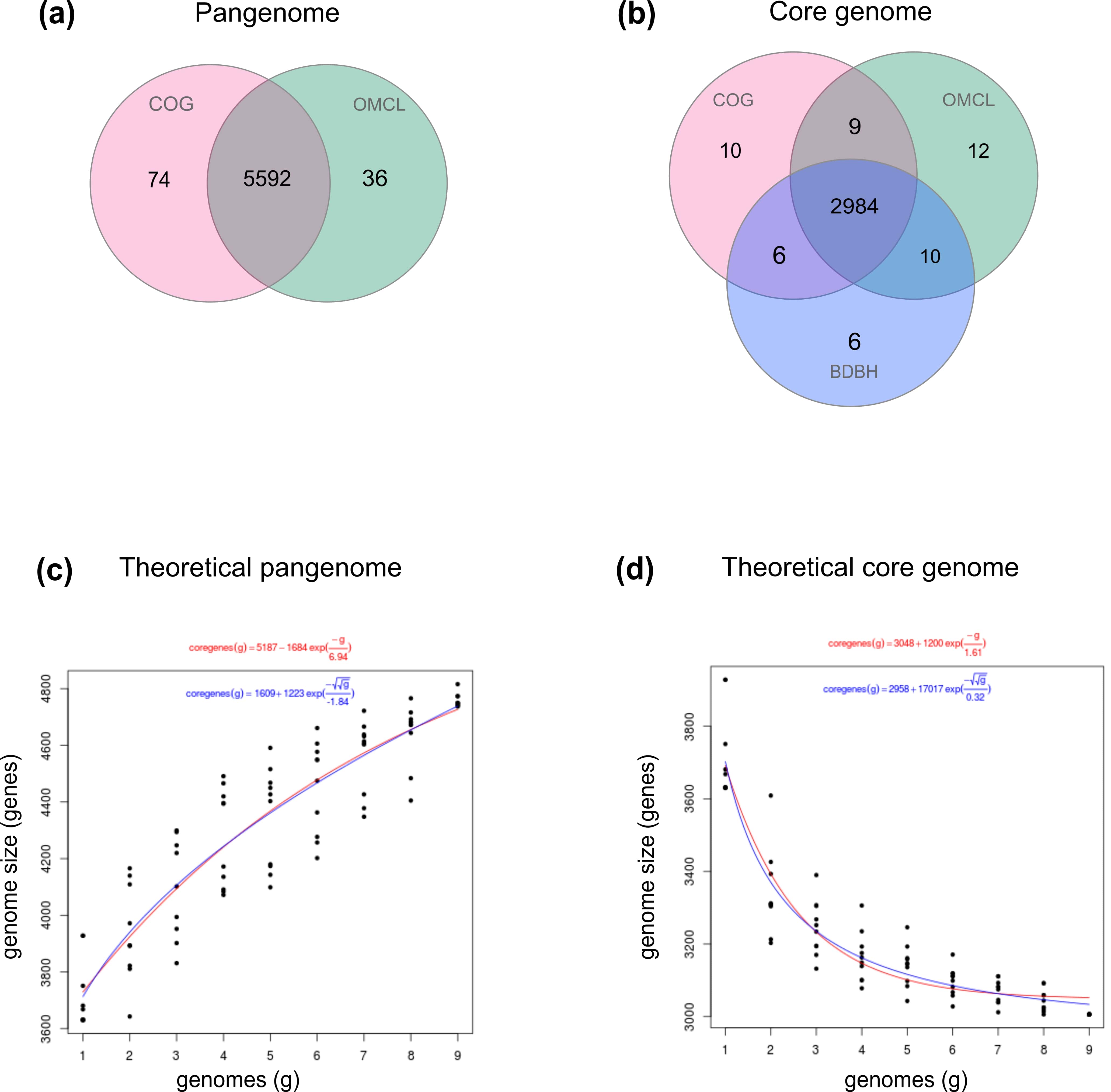

Supplement: Supplementary Figure 2 — Estimation of the core and pangenome sizes: Venn diagrams depicting the (A) pangenome and (B) core genome differentially calculated for the B. pumilus 64-1 and the genomic-related B. pumilus strains by the GET_HOMOLOGUES software; (C) theoretical pangenome size and (D) theoretical core genome size applying the Tettellin (blue) and Willenbrock (red) fits by the OrtoMCL algorithm. [file Image_2.JPEG]

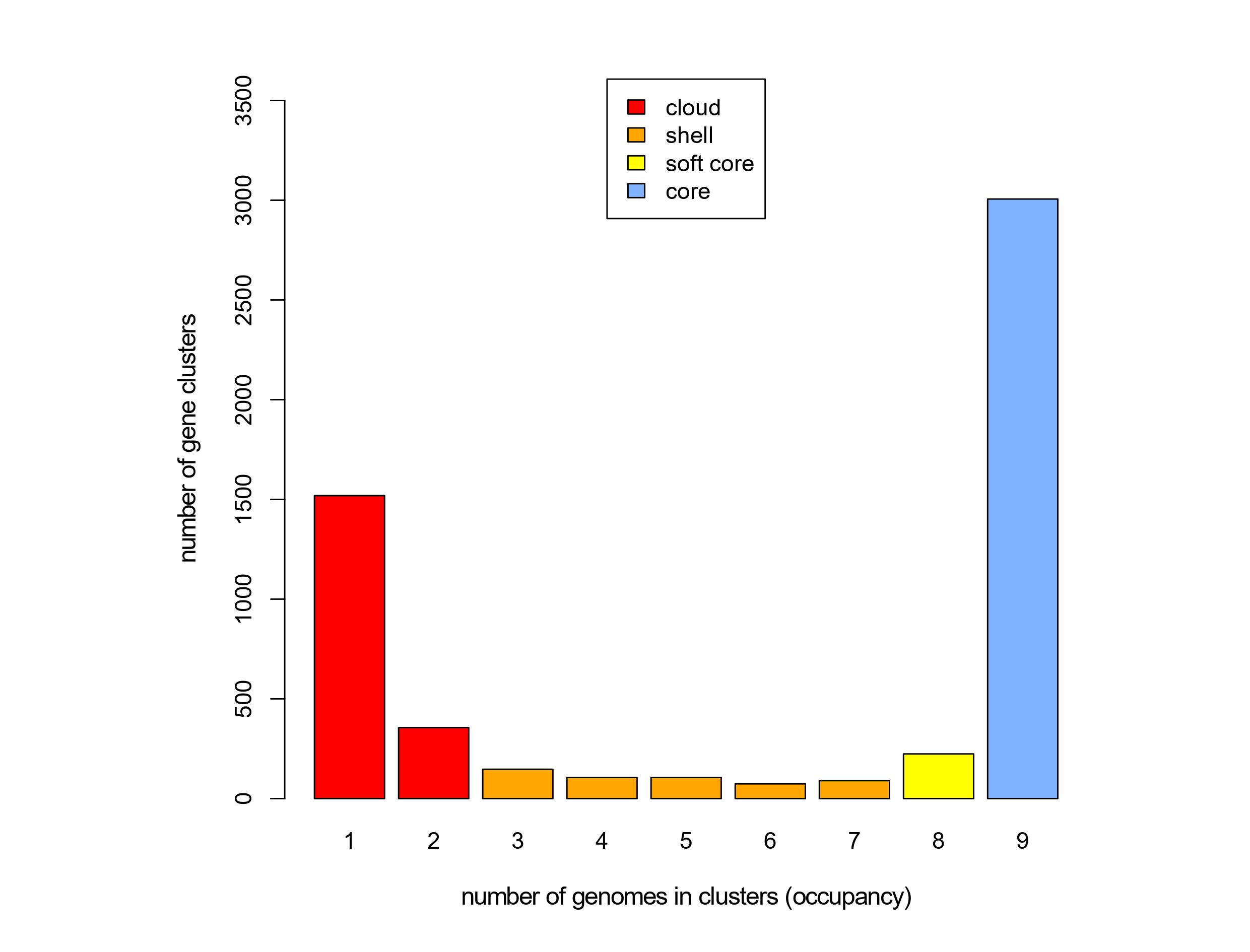

Supplement: Supplementary Figure 3 — Barplot with the frequencies of orthologs clusters assigned to core, soft core, shell and cloud categories according to the orthoMCL algorithm by the GET_HOMOLOGUES software. [file Image_3.JPEG]

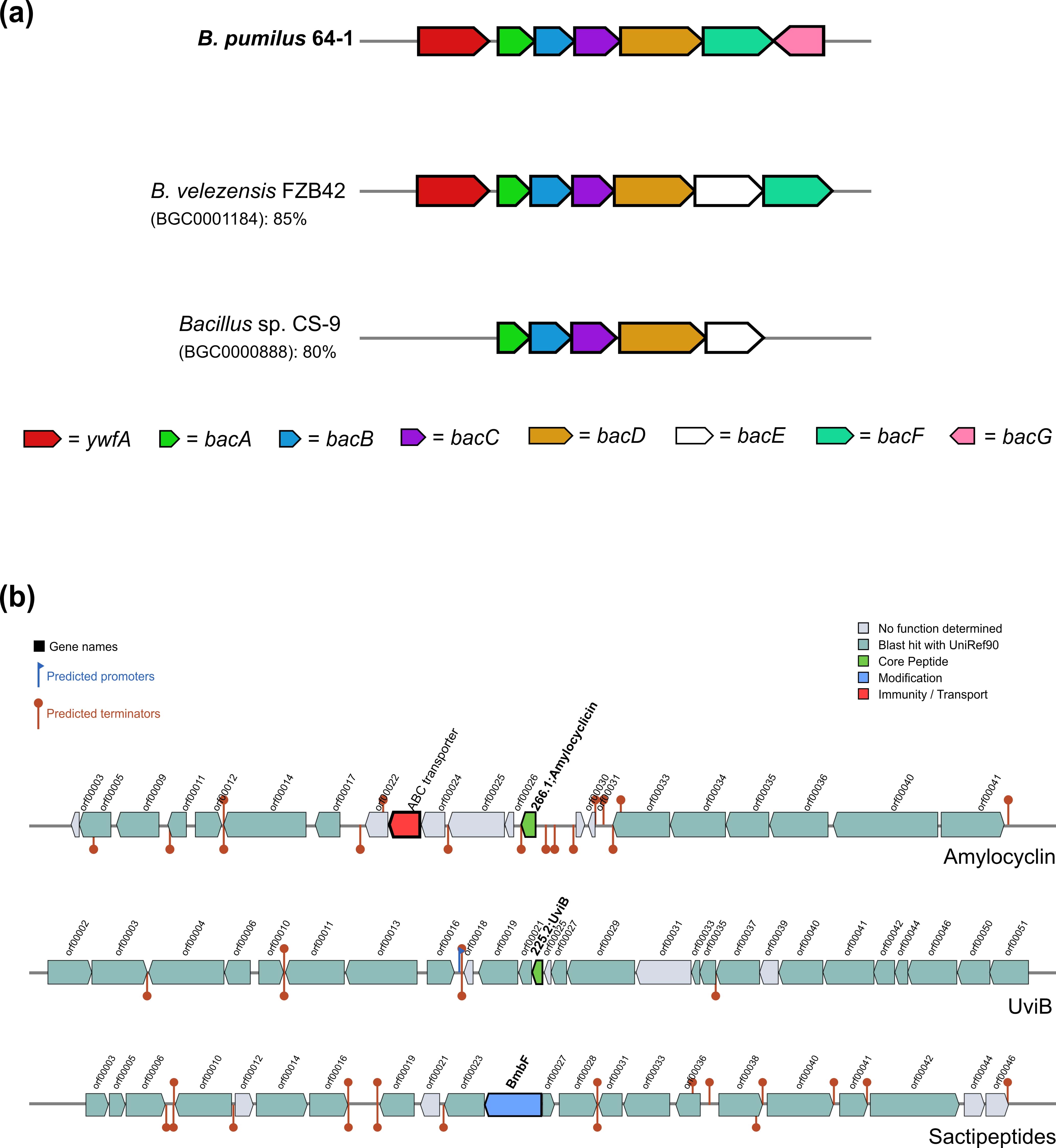

Supplement: Supplementary Figure 4 — Genomic architecture of the: (A) bacilysin gene cluster and its closest BGCs matches (MiBIG accession numbers parenthetical) according to the antiSMASH tool; (B) bioactive peptides-encoding genes within the areas of interest (AOIs) detected by the BAGEL4 software. [file Image_4.JPEG]

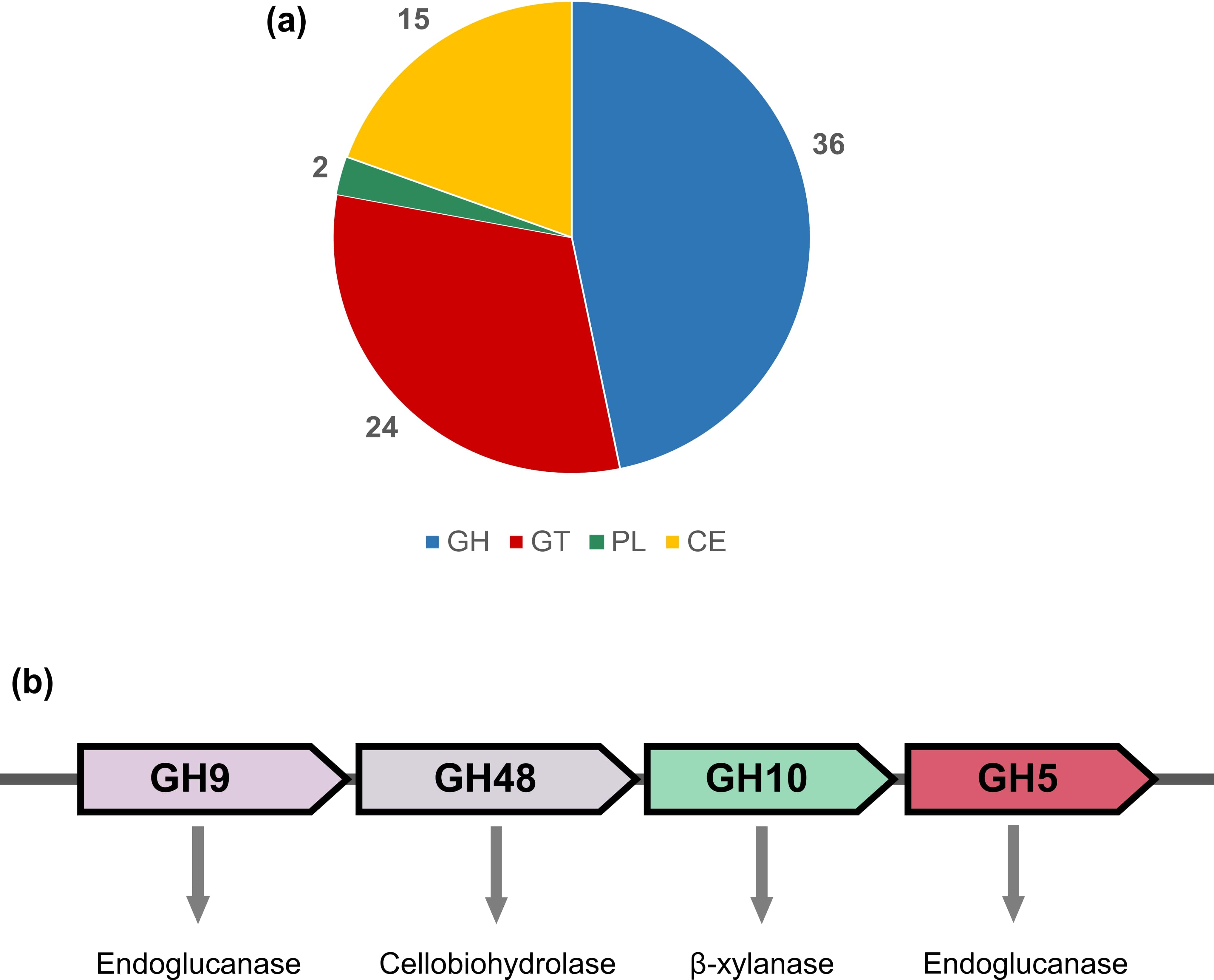

Supplement: Supplementary Figure 5 — Relevant outcomes about the CAZyme repertoire of the B. pumilus 64-1 genome: (A) Distribution of CAZymes classes by the dbCAN 2 metaserver. GHs, glycoside hydrolases; GTs, glicosyltransferases; PLs, polysacharides lyases; CEs, carbohydrate esterases. (B) Concatenated organization of four putative extracellular cellulases (GH9, GH48, and GH5) and xylanase (GH10) genes. [file Image_5.JPEG]

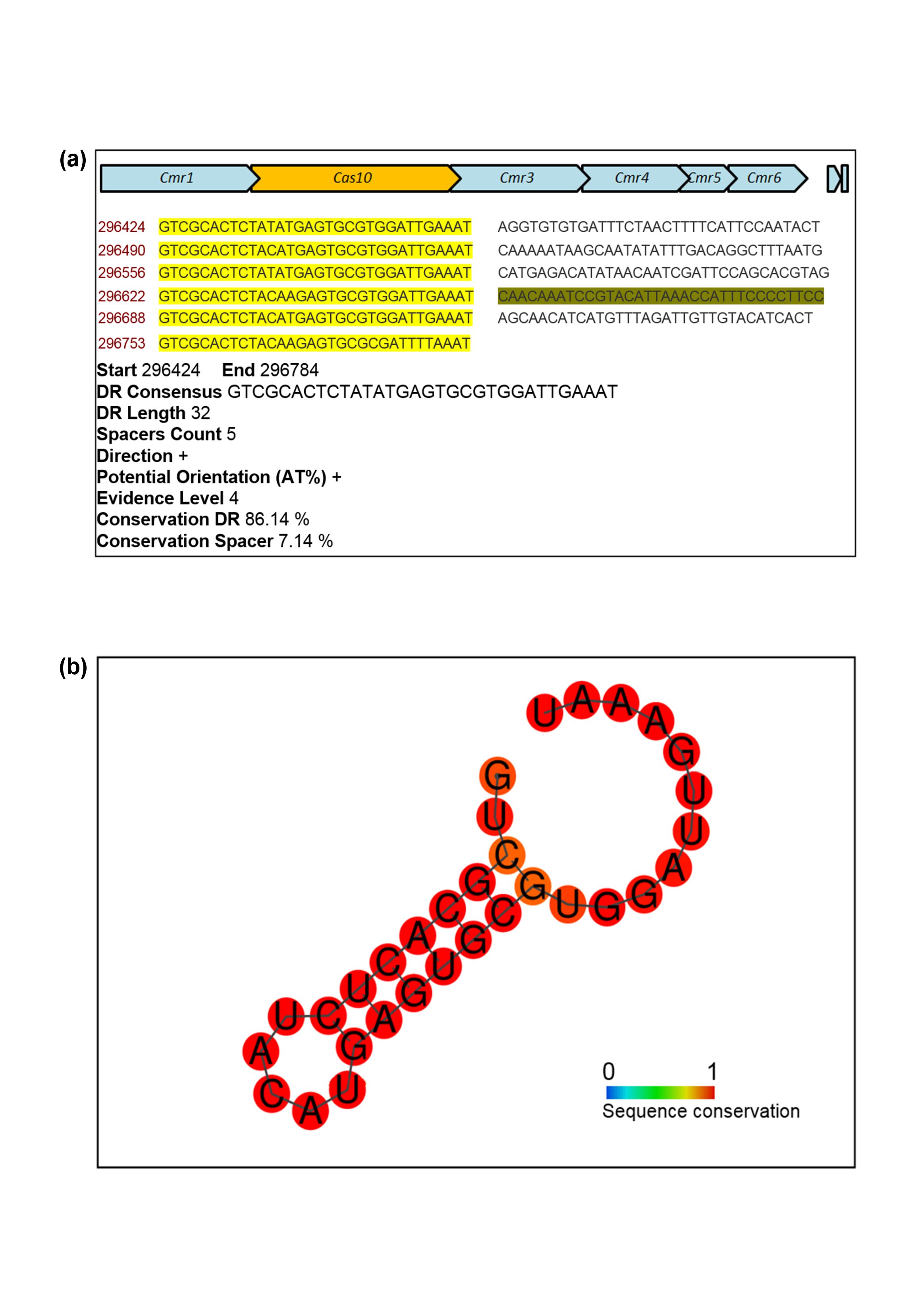

Supplement: Supplementary Figure 6 — Type III-B CRISPR-Cas10 system identified in the B. pumilus 64-1 genome: (A) genomic organization adapted by the CRISPRCasFinder program and Artemis. Highlighted in brown the spacer sequence showing similarity with the B. safensis KCTC 12796BP genome; (B) RNA structure (colored by base-pairing probability) predicted by RNAFolder web server. [file Image_6.JPEG]
