# Supplementary material for: Peeling the Layers Away: The Genomic Characterization of Bacillus pumilus 64-1, an Isolate With Antimicrobial Activity From the Marine Sponge Plakina cyanorosea (Porifera, Homoscleromorpha)
Source: Front Microbiol. 2021 Jan 8;11:592735. doi: 10.3389/fmicb.2020.592735 (PMC7820076; doi:10.3389/fmicb.2020.592735)
Supplement: Supplementary Table 1 — Genome properties of B. pumilus 64-1. [file Table_1.DOCX]

Supplementary Material

**Supplementary Table S1.** Genome properties of *B. pumilus* 64-1

| **Properties** | **Value** | **% Total** |
| --- | --- | --- |
| Assembly size (bp) | 3,667,128 | 100.00 |
| Number of scaffolds | 16 |  |
| Coverage (X) | 190 |  |
| Largest contig (bp) | 951,060 |  |
| N_50_ contig length (bp) | 924,637 |  |
| Completeness (%) | 99.59 |  |
| Contamination (%) | 0.00 |  |
| DNA G+C content (%) | - | 41.5 |
| DNA-coding region (bp) | 3,238,074 | 88.31 |
| rRNA genes | 5 |  |
| tRNA genes | 45 |  |
| tmRNA | 1 |  |
| CDS | 3,705 | 100.00 |
| Genes with signal peptides | 330 | 8.9 |
| Genes with transmembrane segments | 1,011 | 27.28 |
| Subcellular localization of CDS |  |  |
| Cytoplasmic Membrane | 1,061 | 28.63 |
| Cytoplasmic | 1,908 | 51.49 |
| Cell wall | 36 | 0.97 |
| Extracellular | 60 | 1.62 |
| Unknown | 640 | 17.27 |
| CRISPR repeats | 2 |  |

CDS: coding sequences; CRISPR: Regularly Interspaced Short Palindromic Repeats; rRNA: ribosomal RNA; tRNA: transporter RNA; tmRNA: transfer-messenger RNA.
